# Insulin resistance, coronary artery lesion complexity and adverse cardiovascular outcomes in patients with acute coronary syndrome

**DOI:** 10.1186/s12933-024-02276-1

**Published:** 2024-05-16

**Authors:** Qiang Chen, Shiqiang Xiong, Tao Ye, Yanxiang Gao, Jian Wang, Xingliang Li, Yike Li, Caiyan Cui, Hanxiong Liu, Zhen Zhang, Lin Cai, Jingang Zheng

**Affiliations:** 1 Institute of Clinical Medical Sciences, China-Japan Friendship Hospital, Chinese Academy of Medical Sciences, Peking Union Medical College, Beijing, China; 2 Department of Cardiology, The Third People’s Hospital of Chengdu, Affiliated Hospital of Southwest Jiaotong University, Chengdu, Sichuan China; 3 Department of Cardiology, China-Japan Friendship Hospital, Beijing, China

**Keywords:** The triglyceride-glucose index, Acute coronary syndrome, Insulin resistance, Mediation, SYNTAX score

## Abstract

**Background:**

Insulin resistance (IR) is linked to both the complexity of coronary artery lesions and the prognosis of acute coronary syndrome (ACS). However, the precise extent of this correlation and its impact on adverse cardiovascular outcomes in ACS patients remain unclear. Therefore, this study aims to investigate the intricate relationship between IR, coronary artery lesion complexity, and the prognosis of ACS through a cohort design analysis.

**Method:**

A total of 986 patients with ACS who underwent percutaneous coronary intervention (PCI) were included in this analysis. IR was assessed using the triglyceride-glucose (TyG) index, while coronary artery lesion complexity was evaluated using the SYNTAX score. Pearson’s correlation coefficients were utilized to analyze the correlations between variables. The association of the TyG index and SYNTAX score with major adverse cardiovascular events (MACEs) in ACS was investigated using the Kaplan-Meier method, restricted cubic splines (RCS), and adjusted Cox regression. Additionally, a novel 2-stage regression method for survival data was employed in mediation analysis to explore the mediating impact of the SYNTAX score on the association between the TyG index and adverse cardiovascular outcomes, including MACEs and unplanned revascularization.

**Results:**

During a median follow-up of 30.72 months, 167 cases of MACEs were documented, including 66 all-cause deaths (6.69%), 26 nonfatal myocardial infarctions (MIs) (2.64%), and 99 unplanned revascularizations (10.04%). The incidence of MACEs, all-cause death, and unplanned revascularization increased with elevated TyG index and SYNTAX score. Both the TyG index (non-linear, *P* = 0.119) and SYNTAX score (non-linear, *P* = 0.004) displayed a positive dose-response relationship with MACEs, as illustrated by the RCS curve. Following adjustment for multiple factors, both the TyG index and SYNTAX score emerged as significant predictors of MACEs across the total population and various subgroups. Mediation analysis indicated that the SYNTAX score mediated 25.03%, 18.00%, 14.93%, and 11.53% of the correlation between the TyG index and MACEs in different adjusted models, respectively. Similar mediating effects were observed when endpoint was defined as unplanned revascularization.

**Conclusion:**

Elevated baseline TyG index and SYNTAX score were associated with a higher risk of MACEs in ACS. Furthermore, the SYNTAX score partially mediated the relationship between the TyG index and adverse cardiovascular outcomes.

**Supplementary Information:**

The online version contains supplementary material available at 10.1186/s12933-024-02276-1.

## Introduction

The implementation of reperfusion strategies and the improvement of regional coordinated treatment systems have notably decreased acute-phase mortality among patients with acute coronary syndrome (ACS) [1, 2]. However, despite these advancements, the incidence of long-term adverse events following percutaneous coronary intervention (PCI) continues to rise due to population accumulation [3]. Early identification of high-risk patients and the management of relevant risk factors are beneficial for improving the long-term prognosis of ACS patients [4].

Elevated levels of insulin resistance (IR) have been consistently shown to not only correlate with the development and progression of atherosclerotic cardiovascular disease [5–7] but also be connected to a heightened susceptibility to adverse cardiovascular events [8]. The triglyceride-glucose (TyG) index is widely recognized as a non-invasive, cost-effective, and reliable indicator of IR, comparable to the homeostasis model assessment of insulin resistance (HOMA-IR) [9]. Recent studies have shown that an elevated TyG index is independently associated with the development and progression of a range of cardiovascular diseases, such as myocardial infarction [10], coronary artery calcification [11, 12], peripheral artery disease [13], and stroke [14]. Furthermore, it has been noted to be significantly linked to the complexity of coronary artery disease and adverse cardiovascular events following coronary artery revascularization, regardless of the presence of diabetes [15, 16].

Additionally, multiple previous studies have consistently demonstrated a significant correlation between the complexity of coronary artery lesions and the long-term adverse outcomes subsequent to PCI in ACS, encompassing mortality rates and the necessity for repeat revascularization procedures [17, 18]. The SYNTAX (Synergy Between Percutaneous Coronary Intervention) score is a well-established scoring system utilized to assess the complexity of coronary artery disease (CAD) based on various anatomic risk factors evaluated by angiography [19]. Based on our initial research, we have observed that IR, as measured by the TyG index, is independently linked to a greater likelihood of increased coronary anatomical complexity (SYNTAX score > 22) in patients with ACS [15]. Nonetheless, further evidence is required to better comprehend the intricate relationship between IR, coronary artery lesion complexity, and adverse cardiovascular outcomes, contributing to a more thorough insight into IR as a critical prognostic factor for coronary artery disease.

Therefore, this study aimed to investigate the relationship between IR assessed by the TyG index and the complexity of coronary artery lesions evaluated by the SYNTAX score with long-term adverse outcomes following PCI in patients with ACS. Moreover, we examined whether the link between IR and adverse cardiovascular outcomes is partly mediated by the complexity of coronary artery lesions.

## Methods

### Study population

We consecutively enrolled 986 patients hospitalized at the Third People’s Hospital of Chengdu (Sichuan, China) undergoing coronary angiography and diagnosed with ACS from July 2018 to December 2020. Exclusion criteria were as follows: (1) a history of coronary artery bypass grafting (CABG); (2) critical structural heart disease requiring intervention; (3) severe hepatic, respiratory, or renal insufficiency (creatinine clearance < 15 ml/min); (4) advanced hematological or solid tumors with a limited life expectancy; (5) death during hospitalization; (6) incomplete critical medical data exceeding 10%. This study was approved by the ethics committee of the Third People’s Hospital of Chengdu and strictly complied with the Declaration of Helsinki. All participants provided informed consent either in written or oral form.

### Data collection and definitions

Demographic information, medical history, smoking status, and specific medical details were systematically extracted from patients’ electronic health records. The collection included historical health events like prior percutaneous coronary intervention (PCI), chronic obstructive pulmonary disease (COPD), hypertension, diabetes mellitus, stroke, and atrial fibrillation (AF). These historical details were corroborated by medical records following initial self-reports. ACS encompassed conditions such as unstable angina, ST-segment elevation myocardial infarction (STEMI), and non-ST-segment elevation myocardial infarction (NSTEMI), with diagnoses made in accordance with respective guidelines [20]. The identification of diabetes mellitus [21] was based on either the self-reported use of antidiabetic medications or elevated blood glucose readings, characterized by casual blood glucose levels of 11.1mmol/L or higher, fasting blood glucose levels of 7.0mmol/L or higher, or 2-hour postprandial levels exceeding 11.1mmol/L following a 75 g oral glucose tolerance test. Hypertension was identified through a consistent record of blood pressure readings of 140/90mmHg or above, or the ongoing use of antihypertensive medication [22].

Fasting venous blood samples were collected to measure the plasma levels of total cholesterol (TC), triglycerides (TG), LDL-C, HDL-C, fasting blood glucose (FBG), serum creatinine, brain natriuretic peptide (BNP), and cardiac troponin T (cTnT), fibrinogen (Fib), Homocysteine (Hcy), employing standard laboratory methods. The left ventricular ejection fraction (LVEF) was measured using the two-dimensional modified Simpson’s method, providing essential data for evaluating cardiac function.

The TyG index was determined using the formula: ln [TG (mg/dL) ×FBG (mg/dL)/2]. To compute the baseline SYNTAX score (bSS), a web-accessible calculator (http://syntaxscore.com/) was utilized, with two independent evaluators analyzing the preprocedural angiograms without knowledge of initial clinical features and outcomes. In instances of disagreement, a third evaluator’s input was sought to reach a consensus. All data were entered into a dedicated computer database and assessed for quality.

### Follow-up and endpoints

Follow-up assessments were conducted at 1, 3, 6, and12 months post-discharge, and subsequently every 12 months, either through phone calls or in-person clinic visits. Trained professionals documented any clinical events that occurred during follow-up duration. The primary endpoint was major adverse cardiovascular events (MACEs), which encompassed all-cause death, nonfatal myocardial infarction (MI) and unplanned revascularization. Secondary endpoint included cardiac death, unplanned revascularization, and nonfatal stroke. Verification of all clinical endpoints was achieved through review of medical records as needed. Death from any cause was categorized under all-cause death. Unplanned revascularization was defined as ischemia-driven revascularization due to lesion progression or in-stent restenosis during follow-up after the index procedures. The diagnosis of MI and stroke is established in accordance with internationally recognized guidelines.

### Statistical analysis

Continuous variables were characterized by the mean ± SD or the median with interquartile range (IQR), based on the data’s distribution normality. Group differences were analyzed using t-tests or Mann–Whitney U tests accordingly. Categorical variables were summarized as frequencies and percentages, and comparisons between groups were performed using the chi-square (χ^2^) test or Fisher’s exact test. Correlations among variables, such as the TyG index and baseline SYNTAX score, were evaluated using Pearson’s correlation coefficients. Additionally, a heatmap was created to visually represent each correlation coefficient. The incidence of adverse cardiovascular events in different groups was assessed by the Kaplan–Meier method based on the TyG index and SYNTAX score groups, respectively. Discrepancies between groups were evaluated by log-rank tests.

The dose-response association between the TyG index, baseline SYNTAX score, and adverse cardiovascular outcomes in patients with ACS was illustrated through the use of restricted cubic splines (RCS) curve. Cox regression models were employed to assess the relationship between the TyG index, baseline SYNTAX score, and the incidence of adverse cardiovascular outcomes. Hazard ratio (HR) with 95% confidence interval (CI) were calculated within a time-to-event framework. Additionally, several subgroup analyses were conducted to explore whether the predictive utility of the TyG index and baseline SYNTAX score remained consistent across patients with diverse demographic characteristics or comorbidities. To assess the impact of the TyG index (exposure) on MACEs (outcome) via SYNTAX score (mediator), we employed VanderWeele’s two-stage regression approach for survival data [23]. This method involves fitting two distinct regression models: one for the mediator and another for the outcome. By integrating the parameter estimates and standard errors from both models as per VanderWeele’s specified formulas, we derived the mediation effect size. Specifically, we utilized Cox proportional hazards regression to analyze the outcome (MACEs) and linear regression for the mediator (SYNTAX score). The significance of the mediating effect was assessed through the examination of 1000 bootstrap samples. The same approach was used to assess the effect of the TyG index (exposure) mediated by the SYNTAX score (Mediator) on unplanned revascularization (outcome).

In mediation analysis, subgroup analysis, and COX regression analysis, we employed multiple adjusted models, adjusting various covariates independently, to thoroughly evaluate the robustness and reliability of the findings. In all adjusted models, Model I was adjusted for age, sex, BMI, hypertension, diabetes mellitus, smoking, Previous PCI, serum creatinine. Model II was adjusted for age, BMI, serum creatinine, Diuretics, Fib, acute myocardial infarction (AMI), LVEF. Model III was adjusted for age, sex, BMI, hypertension, diabetes mellitus, smoking, Previous PCI, serum creatinine, Diuretics, Fib, AMI, LVEF. All statistical analyses in the present study were performed with SPSS 24.0 (IBM, Armonk, New York), R Programming Language 4.0.2, Stata/MP 16.0 software and MedCalc19.1 (MedCalc software, Belgium). All tests were 2-sided, and *P* < 0.05 was considered statistically significant.

## Results

### Baseline characteristics

**Fig. 1:**
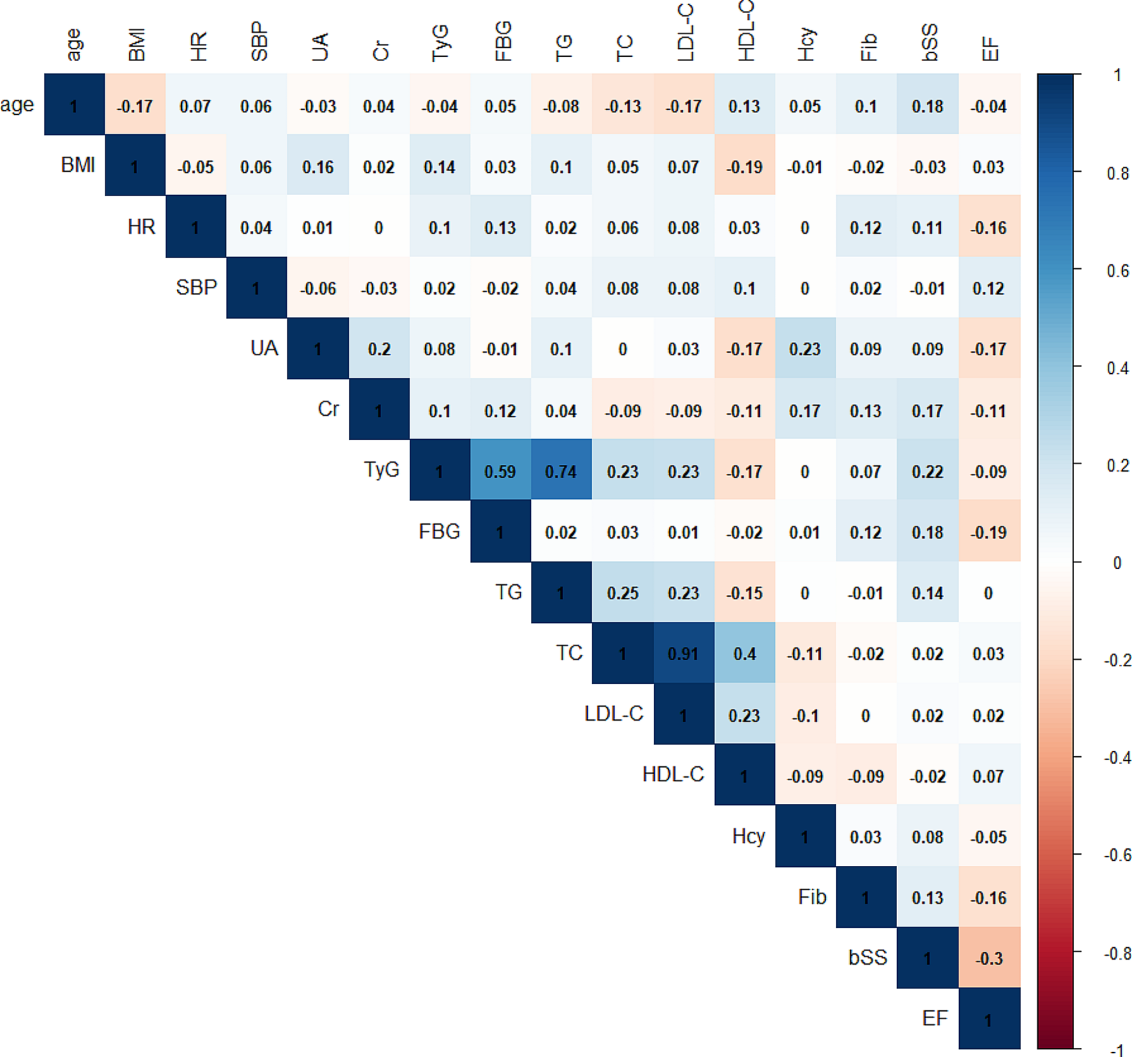
A heatmap illustrating the correlation between different variables**.** BMI, body mass index; SBP, systolic blood pressure; HR, heart rate; UA, Uric acid; FBG, fasting blood glucose; TG, triglyceride; TC, total cholesterol; HDL-C, high density lipoprotein cholesterol; LDL-C, low density lipoprotein cholesterol; Fib, fibrinogen; Hcy, Homocysteine; EF, left ventricular ejection fraction; Cr, Serum creatinine; TyG index, the triglyceride–glucose index; bSS, baseline SYNTAX score. The intensity of color reflects the strength of the correlation

The final cohort consisted of 986 participants who were eligible for the final analysis. The mean age of the population was 66.61 ± 11.42 years. Over a median follow-up period of 30.72 months (interquartile range: 26.13 to 35.07 months), There were 167 (16.94%) cases of MACEs observed, including 66 (6.69%) all-cause deaths, 26 (2.64%) nonfatal MIs, and 99 (10.04%) unplanned revascularizations. Baseline characteristics of the study population are presented in Table 1. Individuals who experienced MACEs were generally older and exhibited higher levels of cTnT, BNP, serum creatinine, uric acid, FBG, TG, Fib, TyG index, and SYNTAX score. They also had lower LVEF, a higher incidence of AMI, and greater usage of insulin and diuretics upon discharge compared to those without MACEs. Furthermore, a heatmap was depicted to visualize the correlations between different variables, revealing a significant positive association between the TyG index and SYNTAX score (*r* = 0.22, *P* < 0.001, Fig. 1).

**Table 1:** Baseline characteristics stratified by the occurrence of MACEs

Variable	Total population	No incident MACEs (*n* = 819)	incident MACEs(*n* = 167)	*P* value
Age, years	66.61 ± 11.42	65.92 ± 11.54	70.03 ± 10.17	< 0.001
Female	279 (28.3)	234 (28.6)	45 (26.9)	0.671
BMI, kg/m^2^	24.32 ± 3.07	24.38 ± 3.08	24.02 ± 3.00	0.191
Smoking, n (%)	541 (54.9)	448 (54.7)	93 (55.7)	0.815
Previous PCI, n (%)	83 (8.4)	67 (8.2)	16 (9.6)	0.553
COPD, n (%)	55 (5.6)	43 (5.3)	12 (7.2)	0.321
Hypertension, n (%)	639 (64.8)	522 (63.7)	117 (70.1)	0.119
Diabetes mellitus	355 (36.0)	288 (35.2)	67 (40.1)	0.224
AF, n (%)	66 (6.7)	51 (6.2)	15 (9.0)	0.194
Previous Stroke, n (%)	75 (7.6)	58 (7.1)	17 (10.2)	0.169
SBP, mmHg	132.23 ± 21.41	132.52 ± 21.35	130.82 ± 21.71	0.350
HR, bpm	77.65 ± 14.80	77.42 ± 14.52	78.77 ± 16.07	0.281
cTnT, pg/ml	37.48 (11.89,863.45)	28.16 (11.33,707.00)	123.20 (15.44,1488.00)	< 0.001
BNP, pg/ml	109.85 (38.28,301.45)	95.20 (35.10,269.60)	167.60 (67.40,645.20)	< 0.001
Serum creatinine, µmol/L	76.45 (64.78,90.80)	76.10 (65.00,89.30)	78.00 (63.10,103.50)	0.042
Uric acid, µmol/L	377.60 ± 104.88	374.48 ± 99.96	392.88 ± 125.47	0.039
FBG, mmol/L	6.94 ± 2.82	7.68 ± 2.69	7.71 ± 3.25	< 0.001
TG, mmol/L	1.60 ± 0.78	1.58 ± 0.78	1.73 ± 0.71	0.017
TC, mmol/L	4.47 ± 1.22	4.48 ± 1.22	4.42 ± 1.23	0.569
HDL-C, mmol/L	1.15 ± 0.30	1.16 ± 0.30	1.13 ± 0.28	0.300
LDL-C, mmol/L	2.77 ± 0.90	2.77 ± 0.89	2.73 ± 0.93	0.562
Hcy, µmol/L	16.62 ± 13.12	16.32 ± 13.39	18.04 ± 11.62	0.122
Fib, g/L	3.88 ± 1.40	3.83 ± 1.34	4.17 ± 1.64	0.004
LVEF	54.91 ± 8.95	55.51 ± 8.50	51.95 ± 10.41	< 0.001
AMI, n (%)	52 1 (52.8)	414 (50.5)	107 (64.1)	0.001
Diagnosis, n (%)				0.001
UA	465 (47.2)	405 (49.5)	60 (35.9)	
NSTEMI	221 (22.4)	168 (20.5)	53 (31.7)	
STEMI	300 (30.4)	246 (30.0)	54 (32.3)	
Aspirin, n (%)	961 (97.5)	803 (98.0)	158 (94.6)	0.010
P_2_Y_12_ receptor inhibitor, n (%)	974 (98.8)	811 (99.0)	163 (97.6)	0.128
Statins, n (%)	959 (97.3)	798 (97.4)	161 (96.4)	0.458
β-blockers, n (%)	687 (69.7)	575 (70.2)	112 (67.1)	0.421
ACEI/ARB, n (%)	416 (42.2)	344 (42.0)	72 (43.1)	0.791
Diuretics, n (%)	151 (15.3)	105 (12.8)	46 (27.5)	< 0.001
Insulin, n (%)	70 (7.1)	52 (6.3)	18 (10.8)	0.042
Oral hypoglycemic agents, n (%)	222 (22.5)	184 (22.5)	38 (22.8)	0.935
TyG index	8.93 ± 0.57	8.89 ± 0.56	9.12 ± 0.59	< 0.001
bSS	13.00 (8.00,20.00)	12.00 (7.00,19.00)	18.00 (12.00,24.50)	< 0.001

Data are presented as mean ± SD, median (IQR) or n (%). BMI, body mass index; PCI, percutaneous coronary intervention; COPD, chronic obstructive pulmonary disease; AF, atrial fibrillation; SBP, systolic blood pressure; HR, heart rate; cTnT, cardiac troponin T; BNP, brain natriuretic peptide; FBG, fasting blood glucose; TG, triglyceride; TC, total cholesterol; HDL-C, high density lipoprotein cholesterol; LDL-C, low density lipoprotein cholesterol; Fib, fibrinogen; Hcy, Homocysteine; LVEF, left ventricular ejection fraction; AMI, acute myocardial infarction; UA, unstable angina; STEMI, ST-segment elevation myocardial infarction; NSTEMI, non-ST-segment elevation myocardial infarction; ACEI/ARB, angiotensin converting enzyme inhibitor/angiotensin receptor blocker; TyG index, the triglyceride–glucose index; bSS, baseline SYNTAX score; MACEs, major adverse cardiovascular events

### Association between the TyG index, SYNTAX score, and the incidence of MACEs

**Fig. 2:**
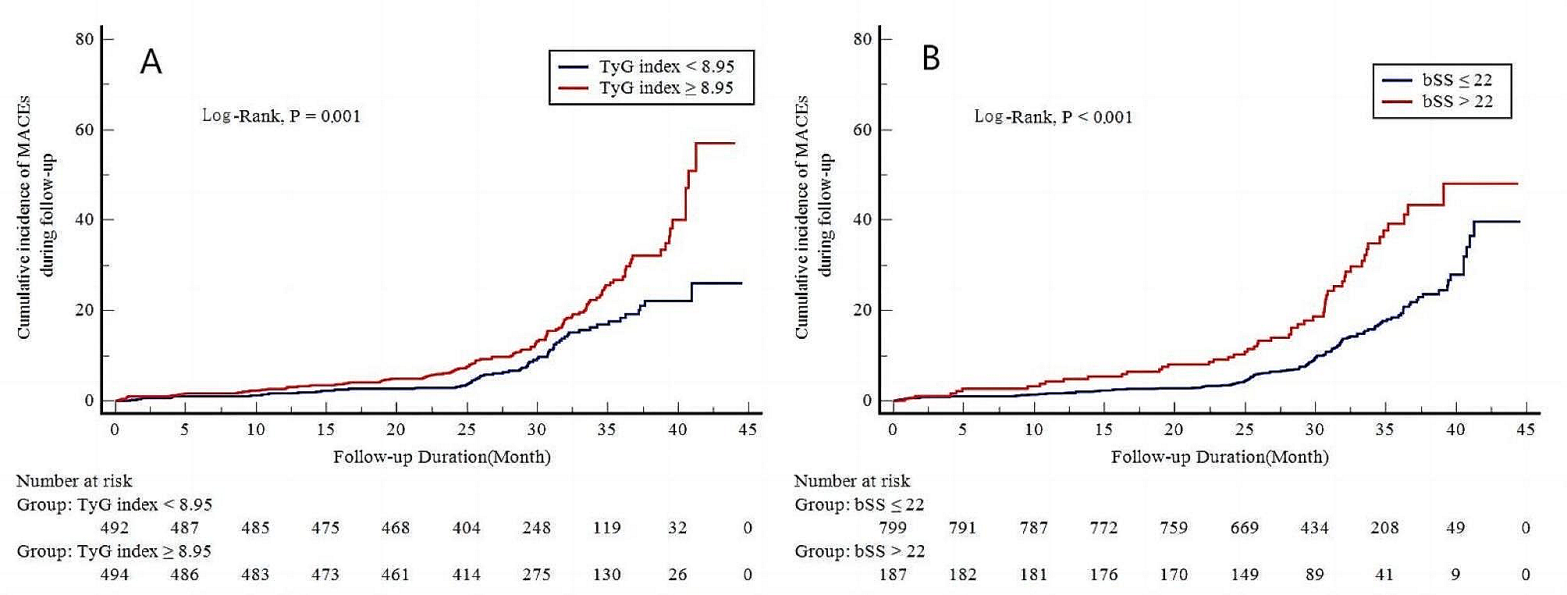
Cumulative incidence of MACEs during follow-up stratified by the TyG index value (**A**) and SYNTAX score (**B**)**.** TyG index, the triglyceride–glucose index; bSS, baseline SYNTAX score; MACEs, major adverse cardiovascular events

Patients were categorized into low-risk (SYNTAX score ≤ 22) and medium/high-risk (SYNTAX score > 22) groups based on the SYNTAX score. Simultaneously, patients were divided into a lower TyG index group (TyG index < 8.95) and a higher TyG index group (TyG index ≥ 8.95) according to the median value of the TyG index. The incidence of major adverse cardiovascular events (MACEs), all-cause death, and unplanned revascularization increased with elevated TyG index and SYNTAX score (Fig. 2 and Additional File: Table S1, Figure S1).

**Fig. 3:**
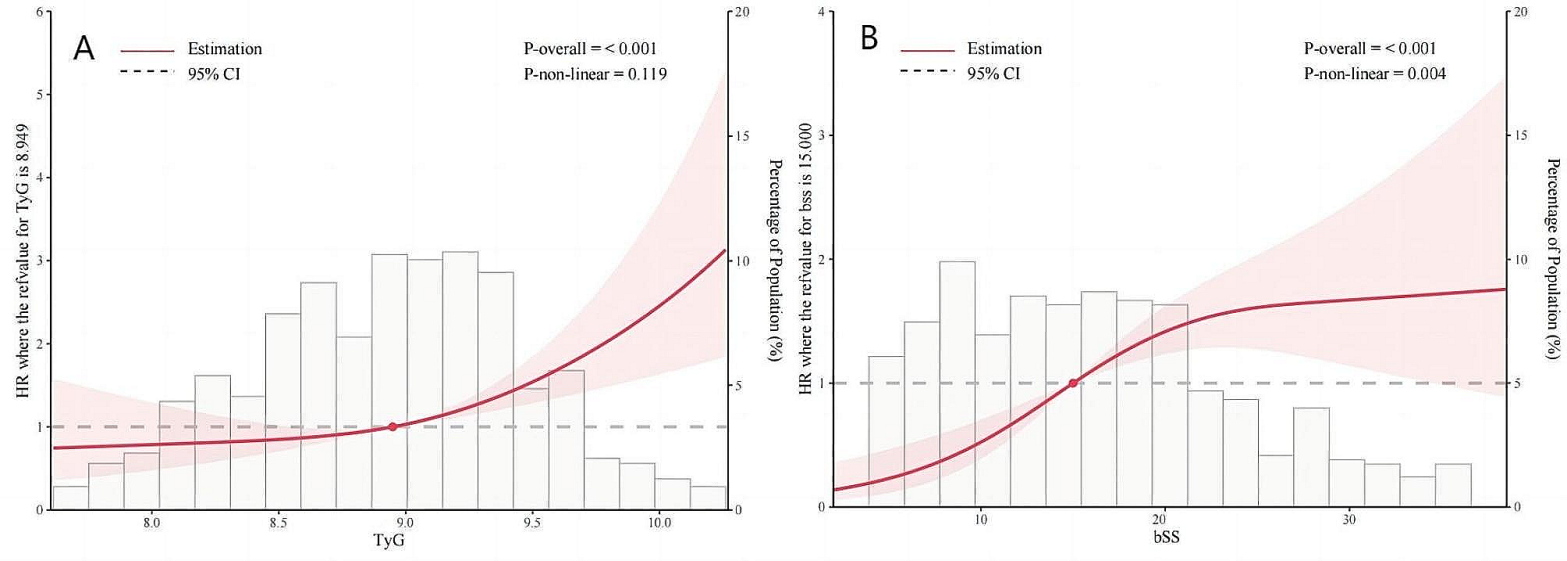
Dose-responsive relationship of the TyG index (**A**) and SYNTAX score (**B**) with the risk of with MACEs in ACS undergoing PCI. TyG index, the triglyceride–glucose index; bSS, baseline SYNTAX score; HR, hazard ratio; CI, confidence interval

Furthermore, both the TyG index (non-linear, *P* = 0.119) and SYNTAX score (non-linear, *P* = 0.004) displayed a positive dose-response relationship with MACEs when analyzed as continuous variables, as depicted by the restricted cubic spline (RCS) curve (Fig. 3). Univariate COX regression showed that the TyG index, age, BMI, AMI, serum creatinine, FBG, TG, Fib, SYNTAX score, LVEF, diuretics, and insulin were risk factors for incidence of MACEs (Additional File: Table S2). The TyG index and SYNTAX score were included as continuous variables in multivariate Cox regression analysis. After adjusting for multiple confounding factors, both an elevated TyG index (Model I: HR 1.9024, 95% CI: 1.3803–2.6219, *P* = 0.0001; Model II: HR 1.6682, 95% CI: 1.2682–2.1944, *P* = 0.0003; Model III: HR 1.9674, 95% CI: 1.4346–2.6979, *P* = 0.0001) and SYNTAX score (Model I: HR 1.0342, 95% CI: 1.0174–1.0512, *P* = 0.0001; Model II: HR 1.0269, 95% CI: 1.0094–1.0447, *P* = 0.0024; Model III: HR 1.0251, 95% CI: 1.0073–1.0432, *P* = 0.0055) were associated with an increased risk of MACEs in patients with ACS undergoing PCI (Table 2).

**Table 2:** Association of the TyG index and baseline SYNTAX score with the risk of MACEs in ACS undergoing PCI

Variables	TyG index			bSS	
	HR (95% CI)	*P*		HR (95% CI)	*P*
Unadjusted	1.8650 (1.4109–2.4653)	< 0.0001		1.0524 (1.0366–1.0684)	< 0.0001
Adjusted Model I	1.9024 (1.3803–2.6219)	0.0001		1.0342 (1.0174–1.0512)	0.0001
Adjusted Model II	1.6682 (1.2682–2.1944)	0.0003		1.0269 (1.0094–1.0447)	0.0024
Adjusted Model III	1.9674 (1.4346–2.6979)	< 0.0001		1.0251 (1.0073–1.0432)	0.0055

TyG index, the triglyceride–glucose index; bSS, baseline SYNTAX score; HR, hazard ratio; CI, confidence interval

Model I was adjusted for age, sex, BMI, hypertension, diabetes mellitus, smoking, Previous PCI, serum creatinine

Model II was adjusted for age, BMI, serum creatinine, Diuretics, Fib, AMI, LVEF

Model III was adjusted for age, sex, BMI, hypertension, diabetes mellitus, smoking, Previous PCI, serum creatinine, Diuretics, Fib, AMI, LVEF

### The predictive value of the TyG index for MACE in various subgroups

**Fig. 4:**
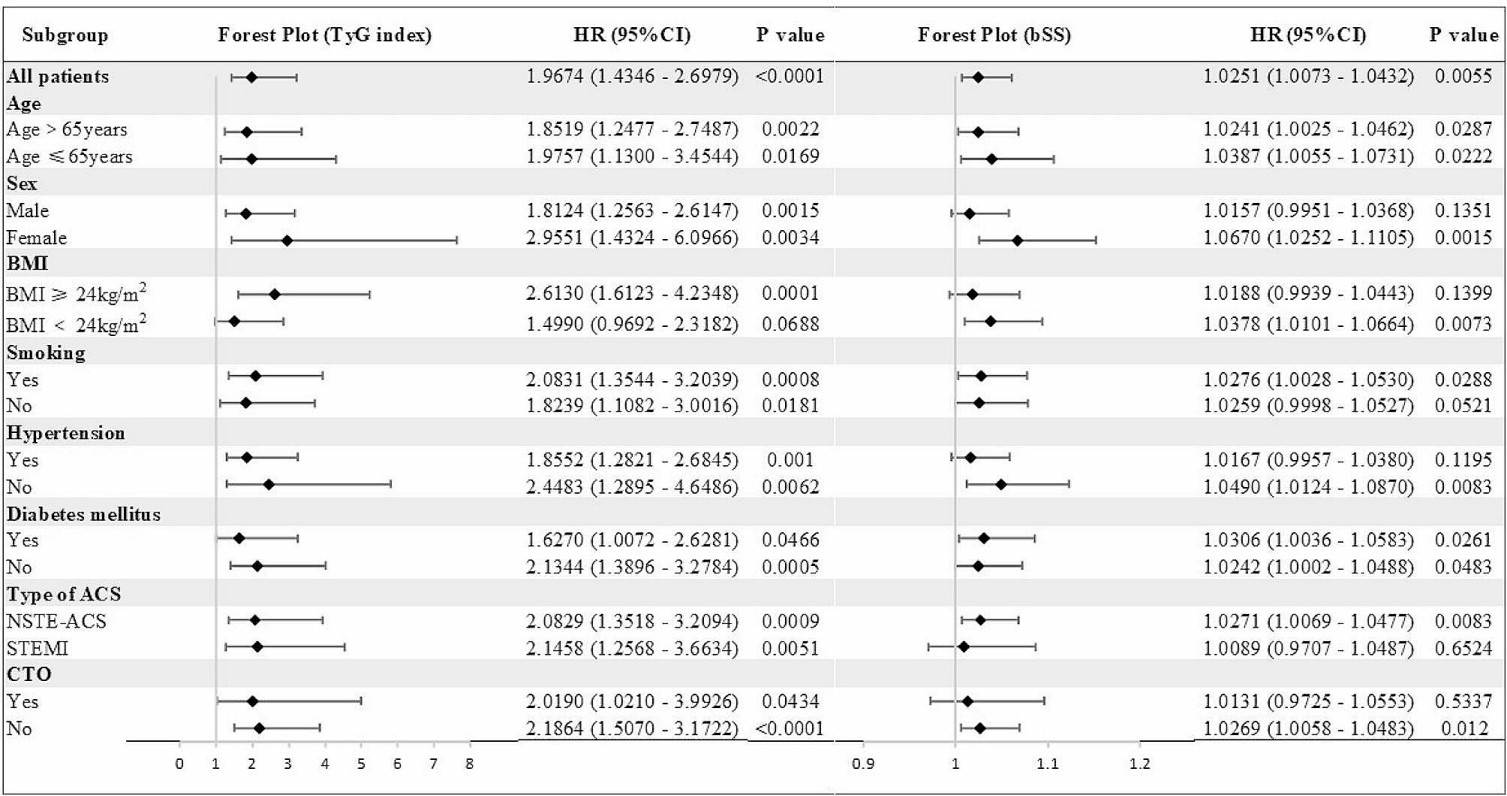
Forest plot illustrating the association of the TyG index and baseline SYNTAX score with the risk of MACEs in ACS undergoing PCI stratified by different subgroups.  TyG index, the triglyceride–glucose index; bSS, baseline SYNTAX score; BMI, body mass index; ACS, acute coronary syndrome; NSTE-ACS, non-ST-segment elevation acute coronary syndrome; STEMI, ST-segment elevation myocardial infarction; CTO, chronic total occlusion; HR was evaluated by 1-point increase of the TyG index and SYNTAX score. HR, hazard ratio; CI, confidence interval. All models were adjusted for age, sex, BMI, hypertension, diabetes mellitus, smoking, Previous PCI, serum creatinine, Diuretics, Fib, AMI, LVEF.

Various subgroup analyses were also conducted to assess whether the predictive value of the TyG index and SYNTAX score remained consistent across diverse demographic characteristics or comorbidities. Figure 4 presents the association between the TyG index and SYNTAX score with MACEs, stratified by age, sex, BMI, diabetes, hypertension, smoking status, and type of ACS. We discovered, after adjusting for multiple factors, that both the TyG index and SYNTAX score emerged as significant predictors of MACEs across various subgroups.

### Mediation analysis

**Fig. 5:**
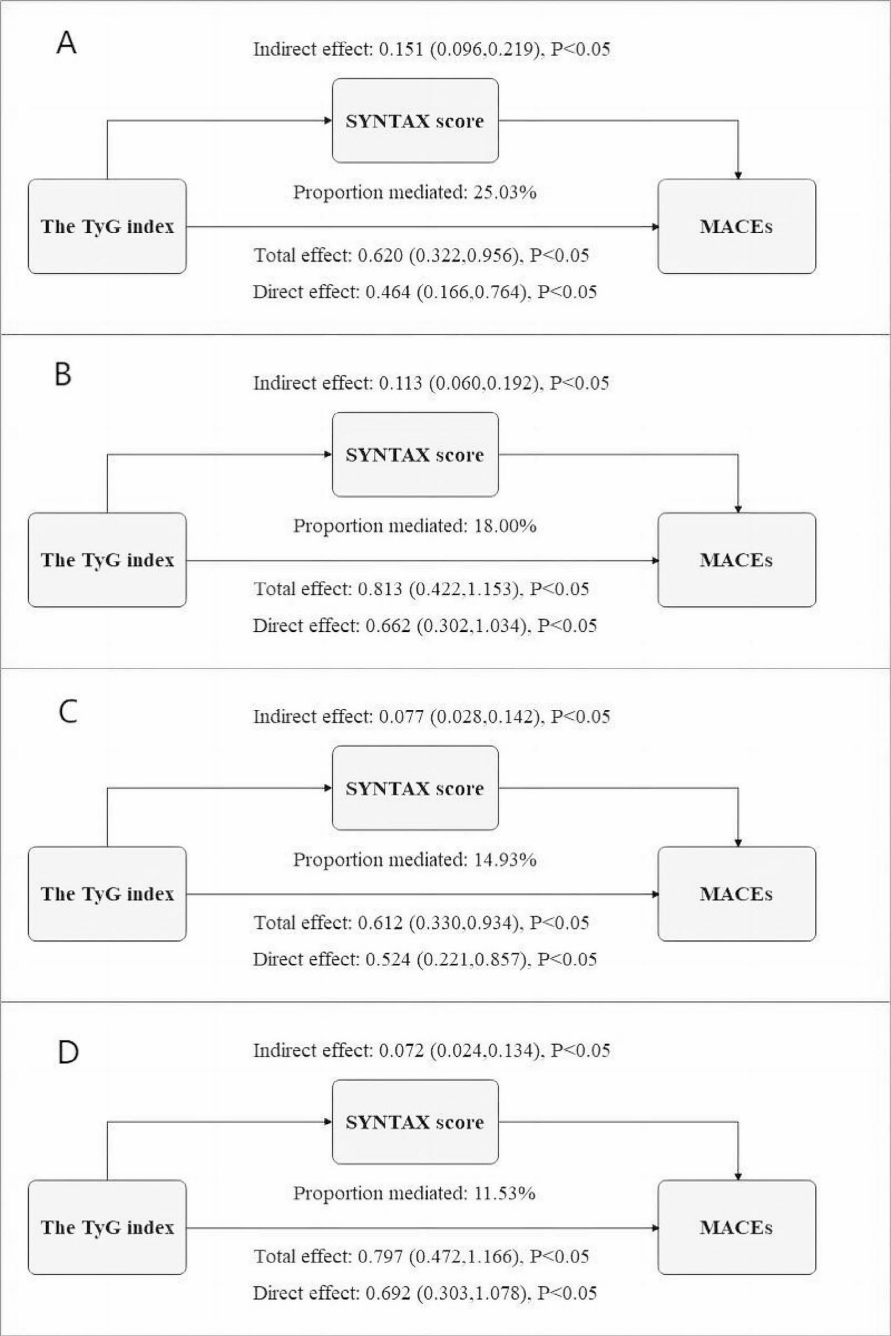
Decomposition of the total association of the TyG index and the risk of MACEs in ACS undergoing PCI into direct and indirect associations mediated by baseline SYNTAX score in different adjusted models, respectively.  TyG index, the triglyceride–glucose index; MACEs, major adverse cardiovascular events; CI, confidence interval; PM, proportion mediated. All effects are presented by β coefficients. A represents the unadjusted Model; B represents the adjusted Model I; C represents the adjusted Model II; D represents the adjusted Model III.  Model I was adjusted for age, sex, BMI, hypertension, diabetes mellitus, smoking, Previous PCI, serum creatinine; Model II was adjusted for age, BMI, serum creatinine, Diuretics, Fib, AMI, LVEF;  Model III was adjusted for age, sex, BMI, hypertension, diabetes mellitus, smoking, Previous PCI, serum creatinine, Diuretics, Fib, AMI, LVEF.

As demonstrated in Table 3; Fig. 5, the mediation analysis revealed that the complexity of coronary artery lesions, as reflected by the SYNTAX score, exerted a significant partial mediating effect on the relationship between IR, as reflected by the TyG index, and the incidence of MACEs across multiple adjusted models. In particular, the mediation proportions of an elevated SYNTAX score were 25.03% (CI: 12.88 − 53.44%, *P*<0.05), 18.00% (CI: 7.94 − 39.84%, *P*<0.05), 14.93% (CI: 4.67 − 36.63%, *P*<0.05), and 11.53% (CI: 2.80 − 28.64%, *P*<0.05) in the unadjusted, adjusted Model I, adjusted Model II, and adjusted Model III analyses, respectively. Moreover, similar mediating effects were observed when cardiovascular adverse events were defined as unplanned revascularization. The result shows that the mediation proportions of an elevated SYNTAX score were 27.18% (CI: 10.03 − 88.50%, *P*<0.05), 17.96% (CI: 5.26 − 48.52%, *P*<0.05), 20.26% (CI: 6.23 − 77.18%, *P*<0.05), and 13.16% (CI: 3.71 − 36.62%, *P*<0.05) in the unadjusted, adjusted Model I, adjusted Model II, and adjusted Model III analyses, respectively (Additional File: Table S3, Figure S2).

**Table 3:** Decomposition of the total association of the TyG index and the risk of MACEs in ACS undergoing PCI into direct and indirect associations mediated by baseline SYNTAX score

Exposures	Association						PM, %	
	Total effect		Indirect effect		Direct effect			
	HR (95% CI)	*P*	HR (95% CI)	*P*	HR (95% CI)	*P*	HR (95% CI)	*P*
Unadjusted	1.865 (1.585,2.465)	< 0.05	1.161 (1.088,1.277)	< 0.05	1.592 (1.198,2.116)	< 0.05	25.03 (12.88,53.44)	< 0.05
Model I	2.225 (1.585,3.137)	< 0.05	1.118 (1.058,1.213)	< 0.05	1.916 (1.351,2.749)	< 0.05	18.00 (7.94,39.84)	< 0.05
Model II	1.867 (1.366,2.544)	< 0.05	1.083 (1.029,1.143)	< 0.05	1.690 (1.246,2.290)	< 0.05	14.93 (4.67,36.63)	< 0.05
Model III	2.198 (1.523,3.288)	< 0.05	1.072 (1.029,1.158)	< 0.05	2.006 (1.375,2.903)	< 0.05	11.53 (2.80,28.64)	< 0.05

HR, hazard ratio; CI, confidence interval; PM, proportion mediated

Model I was adjusted for age, sex, BMI, hypertension, diabetes mellitus, smoking, Previous PCI, serum creatinine

Model II was adjusted for age, BMI, serum creatinine, Diuretics, Fib, AMI, LVEF

Model III was adjusted for age, sex, BMI, hypertension, diabetes mellitus, smoking, Previous PCI, serum creatinine, Diuretics, Fib, AMI, LVEF

## Discussion

In this retrospective cohort study involving 986 individuals with ACS followed for up to 30.72 months, an significant association was found between elevated baseline TyG index, SYNTAX score, and a higher incidence of MACEs post index PCI. These associations retained their statistical significance even after accounting for established cardiovascular disease risk factors in different models and subgroup analyses. Furthermore, the study indicated that the elevated SYNTAX score partly mediated the connection between the TyG index and adverse cardiovascular outcomes, including MACEs and unplanned revascularization, in ACS patient following PCI.

IR, assessed by the homeostasis model assessment of insulin resistance (HOMA-IR), is a metabolic disorder significantly associated with the initiation and advancement of atherosclerosis and cardiovascular disease [5–7]. This condition results in elevated blood glucose levels and excessive insulin secretion, potentially triggering abnormal inflammation and lipid metabolism, thus accelerating atherosclerosis development [24]. In patients with normal glucose tolerance and coronary artery disease, IR, as evaluated by HOMA-IR, is associated not only with the severity of coronary artery disease [25] but also with restenosis and the need for overall new PCI [26]. Numerous studies have validated the TyG index as a direct and cost-effective method for evaluating IR levels, serving as a substitute for HOMA-IR [9]. It is associated with cardiovascular prognosis in various conditions, such as myocardial infarction [16], stroke [14], type 2 diabetes mellitus [27], fatty liver [28], metabolic syndrome [28], and other diseases. Additionally, previous research has elucidated the relationship between the TyG index and long-term adverse cardiovascular events, as well as in-stent restenosis and recurrent revascularization following PCI [29–31]. In line with existing studies, our research illustrated an independent association between a higher TyG index and MACEs in different models and subgroup analyses. The analysis of the restricted cubic spline (RCS) curve revealed a positive dose-response relationship between the TyG index and MACEs, indicating that the incidence of MACEs rises with increasing TyG index levels.

Prior research has shown that IR impacts cardiovascular outcomes by enhancing the advancement of atherosclerosis [6], arterial calcification [11], renal dysfunction [32], inflammation [33], and other mechanisms. The complexity of coronary artery lesions is a strong predictor of adverse cardiovascular outcomes and is associated with elevated TyG index levels [15]. Won KB et al. found that the TyG index serves as an independent predictor for the progression of coronary artery calcification, particularly noticeable in individuals initially lacking significant coronary artery calcification [11]. In a study involving 2792 participants, an elevated TyG index was associated with an increased risk of multi-vessel coronary artery disease (CAD) [34]. Our previous research also revealed a significant positive correlation between the TyG index and SYNTAX scores in patients with ACS undergoing coronary angiography [15]. The SYNTAX score is determined through a comprehensive assessment of coronary lesion quantity, severity, and distribution [19]. A high SYNTAX score indicates intricate and severe coronary artery disease, which is linked to a poorer clinical prognosis [17]. Based on these findings, we hypothesize that coronary artery lesion complexity may play a crucial role as a mediator in the relationship between IR and the risk of MACEs following PCI. To our knowledge, this study represents a pioneering analysis examining the mediating role of the SYNTAX score in the association between the TyG index and the long-term risk of adverse cardiovascular events following PCI.

This study elucidates the mediating function of the SYNTAX score in the correlation between the TyG index and adverse cardiovascular outcomes including MACEs and unplanned revascularization following PCI, thereby amalgamating prior evidence into a holistic pathway for informing clinical decision-making. Globally, there has been a consistent rise in the volume of patients receiving coronary intervention. Individuals with coronary heart disease, particularly those with type 2 diabetes mellitus (T2DM) or intricate coronary artery lesions like left main or multi-vessel lesions, maintain a heightened susceptibility to recurring adverse cardiovascular incidents [4, 18]. Hence, prompt recognition of high-risk patients with unfavorable prognoses is imperative. In clinical practice, evaluating IR in patients with coronary artery disease enhances the overall disease assessment and facilitates the development of more effective, personalized treatment and management strategies. Moreover, comprehending the correlation between IR and the severity of coronary artery disease, along with cardiovascular adverse events, enables physicians to better evaluate patient risk and promptly identify and address potential complications.

Besides, even in patients with normal glucose tolerance, IR is still not only associated with the severity of coronary artery disease but also with restenosis and the overall need for new percutaneous coronary interventions. Therefore, proactive interventions targeting IR, including lifestyle interventions [35] and pharmacotherapy (such as metformin [36, 37], GLP-1 receptor agonists [38], SGLT-2 inhibitors [39], and DPP-4 inhibitors [40]), along with comprehensive cardiovascular risk management, are expected to reduce the long-term cardiovascular adverse events in this susceptible population.

### Limitations

While this study provides valuable information, it also poses certain limitations that warrant careful consideration. Primarily, the single-center, observational design restricts our ability to establish a causal link between the TyG index, SYNTAX score, and adverse cardiovascular events following PCI. Additionally, despite adjustments for known cardiac risk factors, the inherent nature of the observational design means that not all confounding variables may have been accounted for, leaving room for potential residual confounding effects. Furthermore, this study did not employ the HOMA-IR to evaluate the level of IR, precluding a comparative analysis between the TyG index and HOMA-IR in our research context. Moreover, aside from its impact on the complexity of coronary artery lesions, IR can provoke inflammatory responses [8], leading to reduced left ventricular systolic function [41] and renal impairment [32], thereby increasing the risk of adverse cardiovascular events following ischemic coronary events. Further investigation is necessary to explore the intricate relationships among these factors in the future. Finally, given that the study’s cohort consisted solely of Chinese patients, the applicability of these findings across different ethnic groups necessitates further validation.

## Conclusion

This study illustrated the significance of the TyG index and SYNTAX score in identifying patients at a higher risk of MACEs following PCI. It also proposed that the complexity of coronary lesions, evaluated by the SYNTAX score, might partly mediate the connection between IR (as indicated by the TyG index) and the probability of adverse cardiovascular outcomes including MACEs and unplanned revascularization. Targeting IR through specific therapies could provide additional advantages in attenuating cardiovascular risks. The results underscore the importance of integrating metabolic and anatomical evaluations to enhance risk stratification in individuals with ACS.

### Electronic supplementary material

Below is the link to the electronic supplementary material.

Supplementary Material 1

## Data Availability

The datasets used and/or analyzed in the study are available from the corresponding author upon reasonable request.
