# Supplementary material for: Insulin resistance, coronary artery lesion complexity and adverse cardiovascular outcomes in patients with acute coronary syndrome
Source: Cardiovasc Diabetol. 2024 May 16;23:172. doi: 10.1186/s12933-024-02276-1 (PMC11100181; doi:10.1186/s12933-024-02276-1)

**Table S1. Comparison of long-term adverse prognosis stratified by TyG index and bSS, respectively**

| Variable | TyG index＜8.95  (n=492) | TyG index≥8.95  (n=494) | P |  | bSS≤22 (n=799) | bSS＞22 (n=187) | P |
| --- | --- | --- | --- | --- | --- | --- | --- |
|  |
| MACEs, n (%) | 61(12.4) | 106(21.5) | ＜0.001 |  | 115(14.4) | 52(27.8) | ＜0.001 |
| All-cause death, n (%) | 21(4.3) | 45(9.1) | 0.002 |  | 42(5.3) | 24(12.8) | ＜0.001 |
| Cardiac death, n (%) | 12(2.4) | 28(5.7) | 0.010 |  | 29(3.6) | 11(5.9) | 0.160 |
| myocardial infarction, n (%) | 10(2.0) | 16(3.2) | 0.237 |  | 20(2.5) | 6(3.2) | 0.588 |
| Unplanned revascularization, n (%) | 37(7.5) | 62(12.6) | 0.009 |  | 71(8.9) | 28(15.0) | 0.013 |
| Stroke, n (%) | 18(3.7) | 19(3.8) | 0.877 |  | 31(3.9) | 6(3.2) | 0.664 |

Patients were grouped based on their SYNTAX score into low-risk (≤22) and medium/high-risk (>22) categories. Simultaneously, they were divided into lower (index < 8.95) and higher (index ≥ 8.95) TyG index groups, determined by the median TyG index value. MACEs indicate major adverse cardiovascular events, defined as a composite of all-cause death, nonfatal myocardial infarction and unplanned revascularization.

Table S2. Univariate Cox regression analysis for predicting MACEs undergoing PCI

| Variables | Univariate analysis | | |
| --- | --- | --- | --- |
|  | HR | 95%CI | P |
| Age | 1.0349 | 1.0204 - 1.0497 | <0.0001 |
| Female | 0.9664 | 0.6863 - 1.3607 | 0.8447 |
| BMI | 0.9394 | 0.8886 - 0.9930 | 0.0273 |
| AMI | 1.7789 | 1.2962 - 2.4415 | 0.0004 |
| Smoking | 1.0514 | 0.7747 - 1.4269 | 0.7477 |
| Previous PCI | 1.0332 | 0.6167 - 1.7311 | 0.9011 |
| Hypertension | 1.3393 | 0.9614 - 1.8657 | 0.0841 |
| Diabetes Mellitus | 1.1627 | 0.8529 - 1.5850 | 0.3404 |
| SBP | 0.9950 | 0.9877 - 1.0025 | 0.1899 |
| Heart rate | 1.0056 | 0.9954 - 1.0159 | 0.2829 |
| Serum creatinine | 1.1577 | 1.0655 - 1.2579 | 0.0005 |
| FBG | 1.0964 | 1.0483 - 1.1466 | 0.0001 |
| TC | 0.9582 | 0.8468 - 1.0843 | 0.4985 |
| TG | 1.2060 | 1.0279 - 1.4150 | 0.0216 |
| HDL-C | 0.7476 | 0.4334 - 1.2897 | 0.2958 |
| LDL-C | 0.9215 | 0.7755 - 1.0950 | 0.3528 |
| Hcy | 1.0058 | 0.9971 - 1.0145 | 0.1910 |
| Fib | 1.1115 | 1.0232 - 1.2073 | 0.0123 |
| bSS | 1.0524 | 1.0366 - 1.0684 | <0.0001 |
| Tyg index | 1.8650 | 1.4109 - 2.4653 | <0.0001 |
| LVEF | 0.9648 | 0.9503 - 0.9794 | <0.0001 |
| β-blockers | 0.8991 | 0.6509 - 1.2418 | 0.5185 |
| Diuretics | 2.2571 | 1.6071 - 3.1700 | <0.0001 |
| ACEI/ARB | 1.0433 | 0.7680 - 1.4174 | 0.7861 |
| Insulin | 1.3882 | 0.8504 - 2.2662 | 0.1896 |

BMI, body mass index; AMI, acute myocardial infarction; PCI, percutaneous coronary intervention; SBP, systolic blood pressure; FBG, fasting blood glucose; TG, triglyceride; TC, total cholesterol; HDL-C, high density lipoprotein cholesterol; LDL-C, low density lipoprotein cholesterol; Fib, fibrinogen; Hcy, Homocysteine; LVEF, left ventricular ejection fraction; ACEI/ARB, angiotensin converting enzyme inhibitor/angiotensin receptor blocker; TyG index, the triglyceride–glucose index; bSS, baseline SYNTAX score; HR, hazard ratio; CI, confidence interval.

**Table S3. Decomposition of the total association of the TyG index and the risk of unplanned revascularization in ACS undergoing PCI into direct and indirect associations mediated by baseline SYNTAX score.**

| Exposures | **Association** | | | | | | **PM, %** | |
| --- | --- | --- | --- | --- | --- | --- | --- | --- |
|  | Total effect | | Indirect effect | | Direct effect | |  |  |
|  | HR (95% CI) | P | HR (95% CI) | P | HR (95% CI) | P | HR (95% CI) | P |
| Unadjusted | 1.646 (1.115,2.483) | <0.05 | 1.158 (1.089,1.245) | <0.05 | 1.442 (0.982,2.119) | ＞0.05 | 27.18 (10.03,88.50) | <0.05 |
| Model 1 | 2.220 (1.426,3.581) | <0.05 | 1.117 (1.043,1.217) | <0.05 | 1.909 (1.177,3.114) | <0.05 | 17.96 (5.26,48.52) | <0.05 |
| Model 2 | 1.693 (1.166,2.511) | <0.05 | 1.122 (1.052,1.217) | <0.05 | 1.509 (1.044,2.181) | <0.05 | 20.26 (6.23,77.18) | <0.05 |
| Model 3 | 2.328 (1.436,3.772) | <0.05 | 1.096 (1.030,1.186) | <0.05 | 2.037 (1.222,3.276) | <0.05 | 13.16 (3.71,36.62) | <0.05 |

HR, hazard ratio; CI, confidence interval; PM, proportion mediated.

Model I was adjusted for age, sex, BMI, hypertension, diabetes mellitus, smoking, Previous PCI, serum creatinine;

Model II was adjusted for age, BMI, serum creatinine, Diuretics, Fib, AMI, LVEF;

Model III was adjusted for age, sex, BMI, hypertension, diabetes mellitus, smoking, Previous PCI, serum creatinine, Diuretics, Fib, AMI, LVEF.

**Figure S1. Cumulative incidence of all cause death, myocardial infarction, cardiac death, unplanned revascularization, and stroke during follow-up stratified by the TyG index value (A) and SYNTAX score (B), respectively.** TyG index, the triglyceride–glucose index; bSS, baseline SYNTAX score; MACEs, major adverse cardiovascular events.


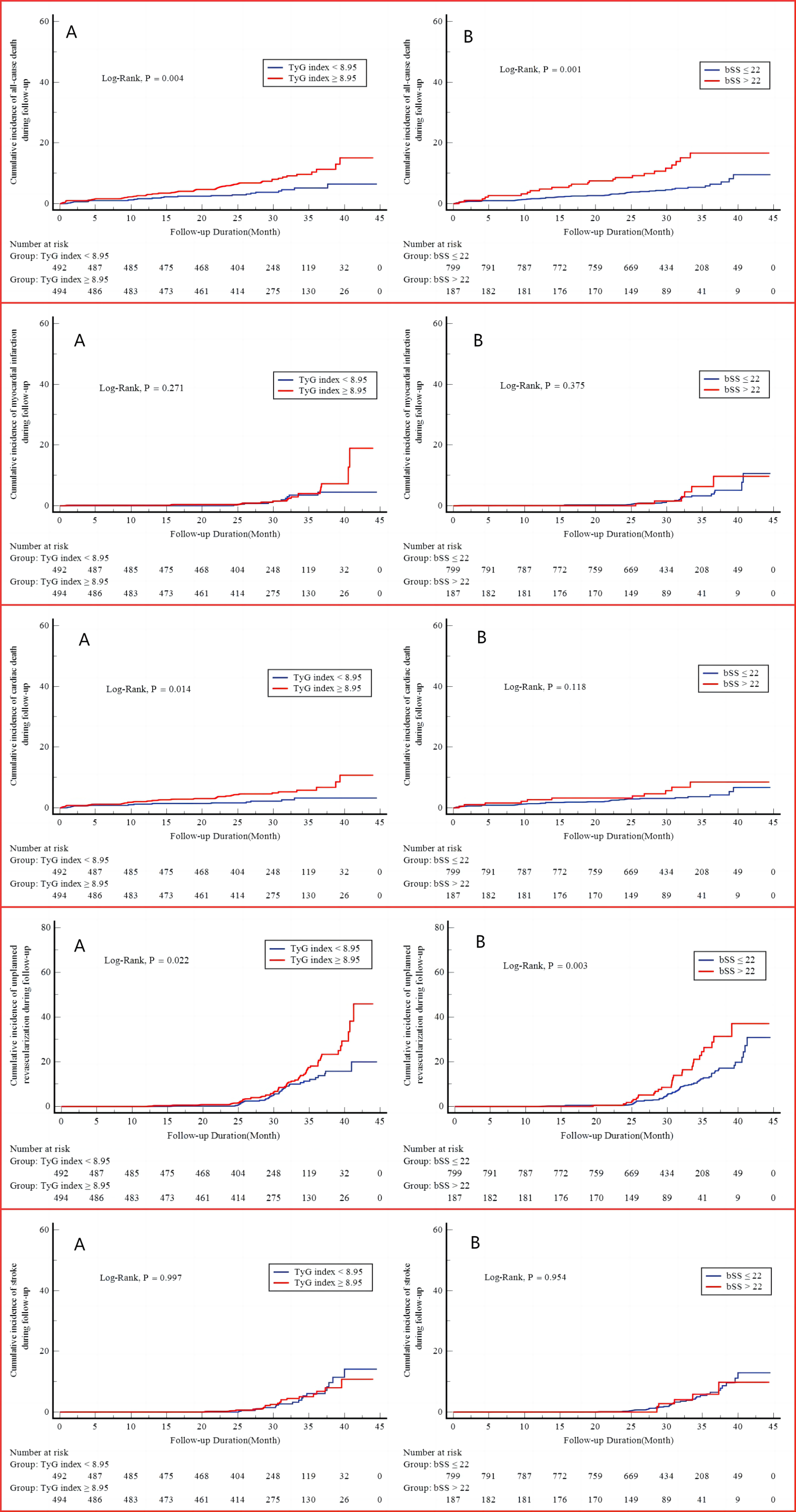


**Figure S2. Decomposition of the total association of the TyG index and the risk of unplanned revascularization in ACS undergoing PCI into direct and indirect associations mediated by baseline SYNTAX score in different adjusted models, respectively.**

TyG index, the triglyceride–glucose index; MACEs, major adverse cardiovascular events; CI, confidence interval; PM, proportion mediated. All effects are presented by β coefficients. A represents the unadjusted Model; B represents the adjusted Model I; C represents the adjusted Model II; D represents the adjusted Model III.

Model I was adjusted for age, sex, BMI, hypertension, diabetes mellitus, smoking, Previous PCI, serum creatinine;

Model II was adjusted for age, BMI, serum creatinine, Diuretics, Fib, AMI, LVEF;

Model III was adjusted for age, sex, BMI, hypertension, diabetes mellitus, smoking, Previous PCI, serum creatinine, Diuretics, Fib, AMI, LVEF.


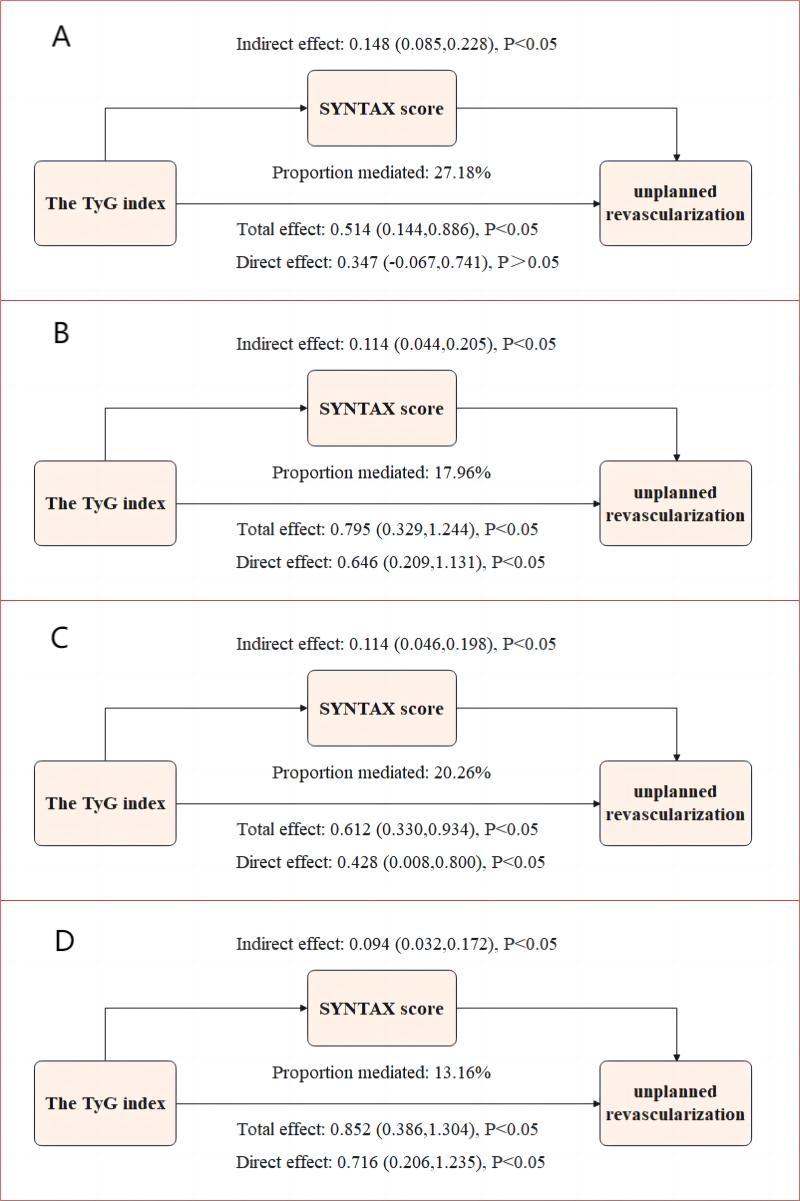

Supplement: Supplementary file 1 — Supplementary Material 1 [file 12933_2024_2276_MOESM1_ESM.doc]
